# Optimal cutoffs of growth discordance for the risk of preeclampsia in twin pregnancies: A single-center retrospective cohort study

**DOI:** 10.3389/fcvm.2022.1073729

**Published:** 2023-01-16

**Authors:** Jie Zhu, Ping An, Huanqiang Zhao, Ying Zhao, Jizi Zhou, Qiongjie Zhou, Xiaotian Li, Yu Xiong

**Affiliations:** ^1^Obstetrics and Gynecology Hospital, Fudan University, Shanghai, China; ^2^The Shanghai Key Laboratory of Female Reproductive Endocrine-Related Diseases, Shanghai, China

**Keywords:** growth discordance, birthweight difference, preeclampsia, twin pregnancy, gestational hypertensive disorders

## Abstract

**Objective:**

To explore the optimal cutoffs of growth discordance for the risk of preeclampsia in twin pregnancies.

**Methods:**

A retrospective cohort study in a university hospital which included twins delivered from February 2013 to September 2020. Restrictive cubic spline (RCS) model was applied to the trend of intertwin birthweight difference (BWD) with the risk of preeclampsia. Logistic regression and subgroup analysis were performed to find the cut-off with statistical significance and clinical meaningfulness.

**Results:**

A total of 2,631 women pregnant with twins were enrolled. RCS showed a nonlinear upward trend of preeclampsia with BWD, and the BWD of 15% was the initial rising point. With the confounders adjusted, only the group with BWD ≥ 25% was found to be significantly associated with an increased risk of preeclampsia (adjusted odds ratio [aOR], 2.44; 95% confidence interval [CI]: 1.74–3.42). Additionally, subgroup analysis showed that both monochorionic (MC) and small for gestational age (SGA) twins were more likely to complicate with preeclampsia.

**Conclusion:**

The growth discordance of 15% during pregnancy may be the preventive point of preeclampsia, and 25% may be the interventional point.

## 1. Introduction

Preeclampsia, which complicates 2–4% of pregnancies globally, is a pregnancy-specific hypertensive syndrome recognized as the leading cause of maternal and fetal morbidity and mortality worldwide (1, 2). Essentially, its preventive strategy is low-dose aspirin prophylaxis, which is initiated at early pregnancy based on the screening for high-risk pregnancy (3). Twin pregnancy is regarded as one of the high-risk factors because it has almost twice the risk of preeclampsia as singleton pregnancy (4–6), and the clinical symptoms tend to develop earlier and grow to be more severe (7). However, some evidence has shown that the prophylactic use of low-dose aspirin does not seem to prevent the occurrence of preeclampsia in twin gestations as effectively as in single pregnancies (8, 9). Therefore, we hypothesize that there might be a unique risk factor for preeclampsia in twins.

As a common complication in twins, growth discordance is defined as intertwin birthweight difference (BWD) of 20% or more based on adverse neonatal outcomes (10–14). Recently, studies have shown that growth discordance is related to the risk of preeclampsia, where growth discordance was also defined as intertwin BWD of 20% or more (15). As intertwin BWD develops over a long period, however, no studies have illuminated the association between growth discordance as a continuous variable and the risk of preeclampsia. In theory, therefore, there must be a dose-response relationship between the intertwin BWD and the risk of preeclampsia, and it is quite necessary to get a deep insight into the correlation before growth discordance is regarded as a specific high-risk factor for preeclampsia in twin pregnancy.

In the current observational study with a diverse population of twin pregnancy, we aimed to discover the optimal cutoffs of the association between growth discordance and the risk of preeclampsia. We tried to describe the trend of the relationship between intertwin BWD and the risk of preeclampsia. Furthermore, we focused on determining the cut-off points of intertwin BWD, from which the risk of preeclampsia would present a rising change, statistically significant and clinically meaningful.

## 2. Materials and methods

### 2.1. Study population

Our retrospective cohort study involved the women with twin pregnancies, who were hospitalized in Obstetrics and Gynecology Hospital of Fudan University, Shanghai of China, from February 2013 to September 2020. All pregnant women had signed an informed consent form. This study was approved by the Ethics Committee of Fudan University (FE21194).

The inclusion criteria were based on the prenatal cards established and the routine prenatal care offered until delivery here at the hospital. The exclusion criteria were as follows: (1) gestational age of less than 24 weeks; (2) intrauterine death of one fetus; (3) complications unique to monochorionic (MC) twins, including twin-to-twin transfusion syndrome (TTTS), twin reversed arterial perfusion sequence (TRAPS), or twin anemia-polycythemia sequence (TAPS); (4) missing information of gestational age at delivery, birthweight or gestational hypertension disorders; or (5) presence of chronic hypertension (CH) or development of preeclampsia superimposed on CH.

### 2.2. Data collection

The following information on maternal characteristics, medical history and pregnancy outcomes was collected: maternal age, gravidity, parity, use of assisted reproductive technology (ART), history of abnormal pregnancy, chorionicity, gestational diabetes mellitus (GDM), gestational age (GA) at delivery, delivery mode, neonatal birthweight, and gestational hypertension (GH). Ultrasonography was used during gestation every 2–3 weeks, starting at 16 weeks of gestation for monochorionic pregnancies and every 3–4 weeks, starting from the anatomy scan (18–22 weeks) for dichorionic pregnancies without complications (10, 16). Chorionicity was assessed based on the presence or absence of the lambda sign and/or two gestational sacs or separate placentas. GA was determined according to LMP, or the crown-rump length in the first trimester, or the head circumference after the larger fetus’s 14 weeks of gestation in the cases of spontaneous conception, and also according to the timing of *in vitro* fertilization for pregnancy conceived *via* assisted reproductive technology. Small for gestational age (SGA) was applied to the newborns whose birthweight was less than the 10th percentile for GA (17, 18).

### 2.3. Clinical definitions and outcomes

The primary outcome was the incidence of preeclampsia, and the secondary referred to the incidence of GH and different types of preeclampsia including severe, mild, and preterm preeclampsia. The intertwin BWD was calculated using the following formula: [(birthweight of larger twin − birthweight of smaller twin)/(birthweight of larger twin) × 100%]. The diagnosis was made of preeclampsia based on the American College of Obstetricians and Gynecologists (ACOG) criteria, where preeclampsia was confirmed as hypertension whose systolic blood pressure ≥ 140 mm Hg and/or diastolic blood pressure ≥ 90 mm Hg on two occasions with at least 4 h apart, and developed for the first time after 20 weeks’ gestation with the new onset of proteinuria or involvement of one of the following organ systems: cerebral or visual symptoms, thrombocytopenia, renal insufficiency, impaired liver function, or pulmonary edema (19). Those who had one of the signs and symptoms or systolic blood pressure ≥ 160 mm Hg or diastolic blood pressure ≥ 100 mm Hg, fell under severe preeclampsia. GH was defined as hypertension that developed for the first time after 20 weeks of gestation, but without evidence of preeclampsia. Additionally, the concept of early preterm and preterm preeclampsia was introduced, which developed before 34 and 37 weeks, respectively, to illustrate the severity of the disease.

### 2.4. Statistical analysis

Statistical analysis was performed based on IBM SPSS Statistics for Windows, version 25.0 (IBM Corp., Armonk, NY, USA).

A comparison was made of the baseline characteristics and outcomes among the four study groups. The continuous variables with normal distributions were presented as the mean ± standard deviation (SD), whereas the categorical data, as n (%). F-test or Welch’s *t*-test was performed for the comparison of the quantitative data, and χ2 test or Fisher’s exact test, for the comparison of the categorical data.

A restrictive cubic spline (RCS) model was developed to establish the dose-response relationship between the intertwin BWD and the odds ratios (ORs) of preeclampsia to find a possible intervention point where the risk of preeclampsia would start to increase.

According to this moving trend, the range before the initial change was regarded as the normal control group, and every 5%-interval thereafter, as a study group. The accordant growth was defined as intertwin BWD < 15%, while the discordant, as BWD ≥ 15%, had three groups of 15–20, 20–25, and ≥25%. Both univariate and multivariate logistic regression analyses were performed, with the adjusting of the risk factors known for preeclampsia, including parity, maternal age, delivery mode, assisted reproductive technology, chorionicity, and gestational diabetes mellitus. ORs and 95% confidence intervals (CIs) were calculated for the association of the intertwin BWD with preeclampsia. *P*-values below 0.05 were considered statistically significant, and ORs above 2.0 were defined as clinically significant (20). To maximize statistical power and minimize bias that might occur if those with missing data were excluded from analyses, the multivariate multiple imputations with chained equations were used to input the missing values (21), and a pooled analysis was made of the five copies of the data comprised on the association of the intertwin BWD and the risk of preeclampsia in the multivariate logistic regression analyses, which were repeated with the complete data cohort for comparison. The supporting information added additional power to the statistical analyses.

We made prespecified exploratory subgroup analyses of preeclampsia to estimate the heterogeneity of intertwin BWD effects between the prespecified subgroups: chorionicity in dichorionic or monochorionic twins and SGA. We performed the subgroup analyses only when there were positive outcomes within each subgroup with different degrees of BWD, while we ran the tests of interaction on all subgroups.

## 3. Results

A total of 2,705 women with twin pregnancies were eligible for the current retrospective analysis, of whom 74 were excluded for their GA of <24 weeks (*n* = 3), intrauterine death of one fetus (*n* = 25), TTTS/TRAPS/TAPS (*n* = 27), missing/incomplete birthweight data (*n* = 4), and CH/preeclampsia superimposed on CH (*n* = 17), hence 2,631 enrolled in the investigation (Supplementary Figure 1). It was found that the characteristics were similar between the cohort of the women enrolled for the main analysis and the larger cohort of the women eligible (Supplementary Table 1).

As indicated in Table 1 with the baseline characteristics of the four groups, there was no significant difference in maternal age, nulliparous, ART, history of abnormal pregnancy, GDM, chorionicity, and delivery mode. As the degree of the intertwin BWD increased, a steady but significant decrease was observed in GA at delivery, average birthweight and smaller-twin birthweight, respectively (*p* < 0.001), except for an increase in the proportion of SGA in one twin (*p* < 0.001).

**TABLE 1:** Baseline characteristics of different degrees of intertwin BWD.

Baseline characteristics	*N*	<15%	15–20%	20–25%	≥25%	*P*-value
Maternal age	2,332	30.9 ± 4.0	30.9 ± 4.3	31.5 ± 4.2	31.1 ± 4.5	0.394
Nulliparous	2,535	1,543 (83.6)	276 (85.4)	153 (79.7)	144 (82.3)	0.373
ART	2,631	982 (51.1)	173 (52.0)	105 (52.8)	92 (51.7)	0.968
History of abnormal pregnancy	2,631	35 (1.8)	4 (1.2)	5 (2.5)	2 (1.1)	0.681
GDM	2,631	383 (19.9)	75 (22.5)	45 (22.6)	37 (20.8)	0.624
Gestational age at delivery (wk)	2,631	35.8 ± 1.9	35.7 ± 2.0	35.5 ± 2.0	34.9 ± 2.2	<0.001
MC twin	2,050	217 (21.2)	63 (23.5)	30 (20.3)	38 (27.7)	0.281
Cesarean delivery	2,625	1,809 (94.4)	317 (95.5)	189 (95.0)	172 (96.6)	0.558
Average birthweight (g)	2,631	2498.7 ± 398.7	2459.2 ± 437.4	2387.9 ± 464.7	2164.5 ± 504.3	<0.001
Larger twin birthweight (g)	2,631	2586.4 ± 416.6	2693.1 ± 479.1	2685.9 ± 522.6	2573.3 ± 574.2	<0.001
Smaller twin birthweight (g)	2,631	2411.0 ± 389.1	2225.4 ± 396.8	2089.9 ± 407.7	1755.7 ± 454.1	<0.001
SGA in at least one twin	2,631	436 (22.7)	151 (45.3)	114 (57.0)	140 (79.1)	<0.001

Analyses based on the complete data for each characteristic; in the table numbers varying by characteristic; all data presented as the mean ± standard deviation or n (%). BWD, birthweight difference; ART, assisted reproductive technology; GDM, gestational diabetes mellitus; MC, monochorionic; SGA, small for gestational age.

**FIGURE 1:**
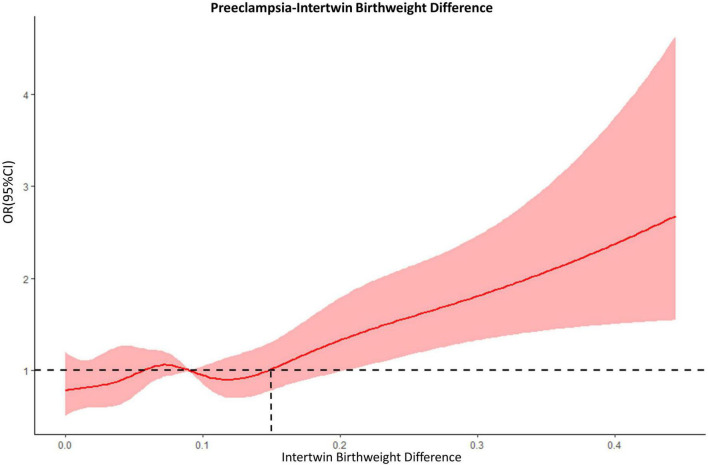
The dose-response relationship between intertwin BWD and preeclampsia. Dashed lines, the reference line (OR = 1.0); red bands, 95% confidential intervals.

The RCS model showed a non-linear relationship between the intertwin BWD and the risk of preeclampsia (Figure 1). A fluctuation was observed in the curve from 0 to 15% in difference, the line of which intersected at around 15%. Given the overlaps between the band of the confidential interval and the reference line, the odds ratio became significant at the BWD ≥ 20%. When an inflection point was set at 15%, therefore, all the participants were divided into four groups according to the degree of BWD: < 15% (1921, 73.0%), 15–20% (333, 12.7%), 20–25% (199, 7.6%), and ≥25% (178, 6.8%).

Of all, there were 518 cases with preeclampsia (19.7%), 244 being severe (9.3%), and 50 delivered before 34 weeks of gestation (1.9%; Table 2). A higher incidence of preeclampsia was found in the group of BWD ≥ 25% than in the group of BWD < 15% (34.3 vs. 17.8%; *p* < 0.001), and the same was true of mild preeclampsia (20.2 vs. 9.8%; *p* < 0.001), of severe preeclampsia (14.0 vs. 8.0%; *p* = 0.005), of preeclampsia with early preterm birth (8.4% vs. 1.2%; *p* < 0.001), and of preeclampsia with preterm birth (25.8 vs. 11.9%; *p* < 0.001). As indicated by the comparison between the group of 20–25% BWD and the control group, a significant increase was observed only in the incidence of severe preeclampsia and preeclampsia with preterm birth, while no significant difference was found in other outcome indicators.

**TABLE 2:** Maternal GH-PE outcomes of different degrees of intertwin BWD.

Outcomes	Accordant	Discordant					
	<15% *n* = 1,921	15–20% *n* = 333	*P*-value	20–25% *n* = 199	*P*-value	≥25% *n* = 178	*P*-value
GH-PE	431 (22.4)	80 (24.0)	0.523	61 (30.7)	0.009	73 (41.0)	0.000
GH	90 (4.7)	12 (3.6)	0.381	15 (7.5)	0.077	14 (7.9)	0.061
PE	342 (17.8)	68 (20.4)	0.253	47 (23.6)	0.044	61 (34.3)	0.000
mPE	189 (9.8)	31 (9.3)	0.764	18 (9.0)	0.720	36 (20.2)	0.000
sPE	153 (8.0)	37 (11.1)	0.056	29 (14.6)	0.002	25 (14.0)	0.005
<34w PE	23 (1.2)	9 (2.7)	0.058	3 (1.5)	0.968	15 (8.4)	0.000
<37w PE	228 (11.9)	44 (13.2)	0.487	37 (18.6)	0.006	46 (25.8)	0.000

All data presented as the mean ± standard deviation or n (%); BWD < 15% group taken as reference. BWD, birthweight difference; GH, gestational hypertension; PE, preeclampsia; mPE, mild preeclampsia; sPE, severe preeclampsia; <34w PE, preeclampsia delivered before 34 weeks; <37w PE, preeclampsia delivered before 37 weeks.

Crude logistic regression analysis showed a significantly increased risk of preeclampsia in the group of 20–25% BWD (OR: 1.43; 95% CI: 1.01–2.02; *p* = 0.045) and in the group of BWD ≥ 25% (OR: 2.41; 95% CI: 1.73–3.35; *p* < 0.001), respectively, when compared with the group of BWD < 15%. With such confounders adjusted as parity, maternal age, delivery mode, ART, chorionicity, and GDM, a significantly increased risk of preeclampsia was only observed in the group of BWD ≥ 25% (adjusted OR [aOR]:2.44; 95% CI:1.74–3.42; *p* < 0.001). As to the association of growth discordance with the risk of mild preeclampsia, preeclampsia with early preterm birth and preterm birth, a significant increase was also observed only in the group of BWD ≥ 25% (Table 3).

**TABLE 3:** The aORs of maternal GH-PE outcomes of different degrees of intertwin BWD.

Outcomes	Crude OR (95% CI)	*P*-value	Adjusted OR (95% CI)	*P*-value
**GH-PE**				
<15%	Reference	–	Reference	–
15–20%	1.09 (0.83–1.44)	0.523	1.09 (0.83–1.43)	0.547
20–25%	1.53 (1.11–2.10)	0.009	1.50 (1.08–2.07)	0.015
≥25%	2.40 (1.75–3.30)	<0.001	2.45 (1.77–3.38)	<0.001
**GH**				
<15%	Reference	–	Reference	–
15–20%	0.76 (0.41–1.41)	0.382	0.76 (0.41–1.41)	0.390
20–25%	1.66 (0.94–2.93)	0.080	1.61 (0.91–2.85)	0.101
≥25%	1.74 (0.97–3.12)	0.065	1.78 (0.99–3.21)	0.054
**PE**				
<15%	Reference	–	Reference	–
15–20%	1.19 (0.89–1.59)	0.253	1.18 (0.88–1.58)	0.274
20–25%	1.43 (1.01–2.02)	0.045	1.40 (0.99–1.99)	0.059
≥25%	2.41 (1.73–3.35)	<0.001	2.44 (1.74–3.42)	<0.001
**mPE**				
<15%	Reference	–	Reference	–
15–20%	0.94 (0.63–1.40)	0.764	0.93 (0.62–1.39)	0.732
20–25%	0.91 (0.55–1.51)	0.720	0.91 (0.55–1.51)	0.715
≥25%	2.32 (1.57–3.45)	<0.001	2.34 (1.57–3.49)	<0.001
**sPE**				
<15%	Reference	–	Reference	–
15–20%	1.44 (0.99–2.11)	0.058	1.44 (0.98–2.11)	0.062
20–25%	1.97 (1.29–3.02)	0.002	1.90 (1.24–2.93)	0.003
≥25%	1.89 (1.20–2.97)	0.006	1.87 (1.18–2.96)	0.008
**<34w PE**				
<15%	Reference	–	Reference	–
15–20%	2.29 (1.05–5.00)	0.037	2.31 (1.06–5.05)	0.036
20–25%	1.26 (0.37–4.25)	0.706	1.22 (0.36–4.11)	0.750
≥25%	7.59 (3.89–14.82)	<0.001	7.53 (3.79–14.94)	<0.001
**<37w PE**				
<15%	Reference	–	Reference	–
15–20%	1.13 (0.80–1.60)	0.487	1.12 (0.79–1.58)	0.529
20–25%	1.70 (1.16–2.49)	0.007	1.65 (1.12–2.43)	0.011
≥25%	2.59 (1.80–3.72)	<0.001	2.55 (1.76–3.68)	<0.001

Adjusted for parity, maternal age, delivery mode, assisted reproductive technology, chorionicity, and gestational diabetes mellitus; BWD < 15% group taken as reference. BWD, birthweight difference; OR, odds ratio; CI, confidence interval; GH, gestational hypertension; PE, preeclampsia; mPE, mild preeclampsia; sPE, severe preeclampsia; <34w PE, preeclampsia delivered before 34 weeks; <37w PE, preeclampsia delivered before 37 weeks.

Additionally, according to the stratified analyses by SGA and chorionicity (Supplementary Figure 2) to compare the group of BWD ≥ 25% with the group of BWD < 15% in the adjusted model with the incidence of preeclampsia and preeclampsia with preterm birth considered, both MC and SGA twins were found to be more likely to complicate with preeclampsia.

The amount of the missing data ranged from 0 to 22.1% for the different variables; of 2,631 patients, 1,806 (68.6%) had complete data on all variables for the main analyses (Supplementary Table 2). Distributions of any variable with the missing data were the same in the imputation datasets and for the complete case data observed. Logistic regression analyses, which were performed only on the patients who had complete data rendered similar results to those undertaken on the multiple imputed datasets (Supplementary Table 3).

## 4. Discussion

We conducted a large single-center retrospective study on twin pregnancy, for the first time showing the trending of the association of intertwin BWD with the risk of preeclampsia based on the RCS model, and identifying optimal cut-offs. We confirmed the association of BWD with the risk of preeclampsia in a dose-response manner and managed to establish two optimal cut-offs: BWD of 15% as the starting point of the risk of preeclampsia on the move; and BWD of 25% as a threshold of the risk to increase with statistical significance (*p* < 0.05) and clinical meaningfulness (aOR > 2.0), when compared with the accordant twins whose BWD < 15%. Our study might present the possible direction for clinical practice to reduce the incidence of preeclampsia in twin pregnancy considering growth discordance as a potentially high risk. As for the main strength of this study, we regarded the intertwin BWD as a continuous variable and used the RCS model to present the moving trend between BWD and the risk of preeclampsia, thus depicting a non-linear relationship in a real-world retrospective cohort study, characterized by a relatively large sample size. In this model, more importantly, we discovered the theoretical point of 15% to be used to group the cohort logically.

We acknowledge that this study had some limitations. It was of a single-center retrospective study. Despite the potential confounding factors adjusted such as parity, maternal age, ART, chorionicity, and GDM, we were not in a position to eliminate the possible effect of other risk factors such as BMI, renal disease and autoimmune disease, which were not covered in our study. Therefore, further multi-center prospective studies are still needed to powerfully validate our findings. Moreover, we excluded the pregnancies complicated by intrauterine death, which could potentially represent the most severe consequence. Since it was merely an epidemiologic study, additionally, we failed to obtain pathological indicators for our study. Further basic and clinical research is required into the mechanism of intertwin BWD and preeclampsia.

In recent years, several studies have focused on the association between growth discordance and gestational hypertensive disorders (GHD) (15, 22). However, most of the studies set a single cut-off (20 or 25%) of BWD for the definition of growth discordance and were not consistent with each other (23, 24). As a continuous quantitative variable which serves as an ideal clinical marker, growth discordance reflects three sequential statuses: normal, subclinical and clinical phenomena. Using a single cutoff point to distinguish the normal from the abnormal group might miss some important details, such as the marker’s value being close to the cutoff point, which would have allowed patients to be divided into different groups but with little difference. Until now, no studies have illuminated the relationship between growth discordance as a continuous variable and the risk of preeclampsia, thus failing to show the real relationship between the BWD of 20–25% and the incidence of preeclampsia.

In the current study, we considered BWD as a continuous variable and used RCS to present the BWD-associated trending risk of preeclampsia, finding that this trend was a nonlinear model. As the trend of the model showed, BWD of 15% may be the initial rising point of the risk of preeclampsia. We thus defined the accordant growth as intertwin BWD < 15% and found that growth discordance of 25% was a threshold of the risk to increase with statistical significance (*p* < 0.05) and clinical meaningfulness (aOR > 2.0) compared with accordant twins. At present, it is generally accepted that there is heterogeneity in the pathogenesis of preeclampsia, including reduced uteroplacental blood supply, increased fetoplacental demands, or both result in uteroplacental mismatch (25). Twin is regarded as a risk of preeclampsia due to its increased fetoplacental demands. Our study provided new evidence of the underlying pathogenetic mechanism for the significant association of growth discordance with preeclampsia, especially in the subgroup of SGA. True, the development of growth discordance is a long, slow and progressive process after the occurrence of placental malperfusion in the smaller fetus, which is similar to SGA and preeclampsia in singleton pregnancy. Therefore, it was suggested that twins with growth discordance could align with the pathogenesis involving both reduced uteroplacental blood supply and increased fetoplacental demands.

Birthweight, as a gestational endpoint variable, is a precise value. Our study confirmed the association of growth discordance represented by intertwin BWD with the risk of preeclampsia in a dose-response manner and managed to establish two optimal cut-offs. However, during gestation, growth discordance develops gradually over a long period and can only be determined by the difference of estimated fetal weight (EFW) currently. We suppose that when EFW difference during pregnancy is less than 15%, routine health care and monitoring may be enough, based on the risk of twins’ own. When EFW difference is over 15%, it is imperative that rigorous monitoring be implemented, accompanied by increased frequency of antenatal care visits, maternal self-monitoring for hypertension, and monthly maternal laboratory tests for end organ involvement. In this case, the efficacy of low-dose aspirin prophylaxis needs to be further explored. Once EFW difference is over 25%, clinical intervention should be adopted, including timely termination of pregnancy, if the fetuses are mature already. Nevertheless, due to the inevitable error between EFW and actual fetal weight, multi-center prospective studies with serial sonographic assessments of fetal biometry performed by experienced ultrasound physicians are needed to verify the efficacy and effectiveness.

## 5. Conclusion

Our study shows that the optimal cutoffs of growth discordance for the risk of preeclampsia in twin pregnancies seem to be 15 and 25%, respectively. The growth discordance of 15% may be the warning line, across which women with twin pregnancy should be closely monitored, and 25% may be the interventional line, across which prompt and proper intervention is recommended. However, further investigation is still required to support our conclusion.

## Data availability statement

The raw data supporting the conclusions of this article will be made available by the authors, without undue reservation.

## Ethics statement

The studies involving human participants were reviewed and approved by the Ethics Committee of Fudan University. The patients/participants provided their written informed consent to participate in this study.

## Author contributions

YX and XL: design. JZhu, PA, and HZ: planning, conduct, and data analysis. JZhu: manuscript writing. All authors contributed to the article and approved the submitted version.

## Supplementary material

Supplementary Figure 1 Description of the study groups.

Supplementary Figure 2 The aORs of maternal preeclampsia outcomes of intertwin BWD ≥ 25% in subgroup analysis by SGA and chorionicity.
