# Supplementary figures and images for: Optimal cutoffs of growth discordance for the risk of preeclampsia in twin pregnancies: A single-center retrospective cohort study
Source: Front Cardiovasc Med. 2023 Jan 16;9:1073729. doi: 10.3389/fcvm.2022.1073729 (PMC9884673; doi:10.3389/fcvm.2022.1073729)

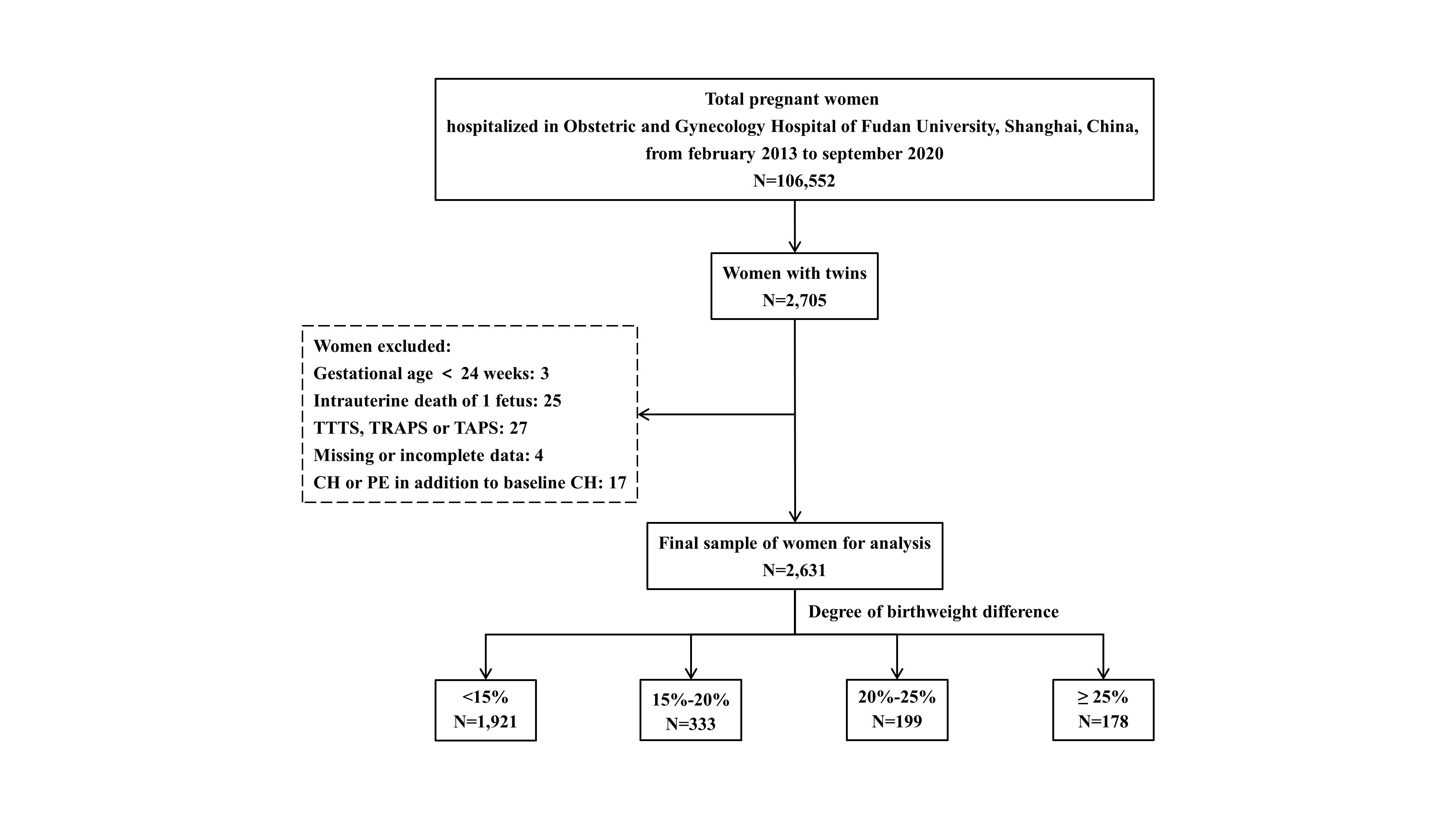

Supplement: Supplementary Figure 1 — Description of the study groups. [file Image_1.JPEG]

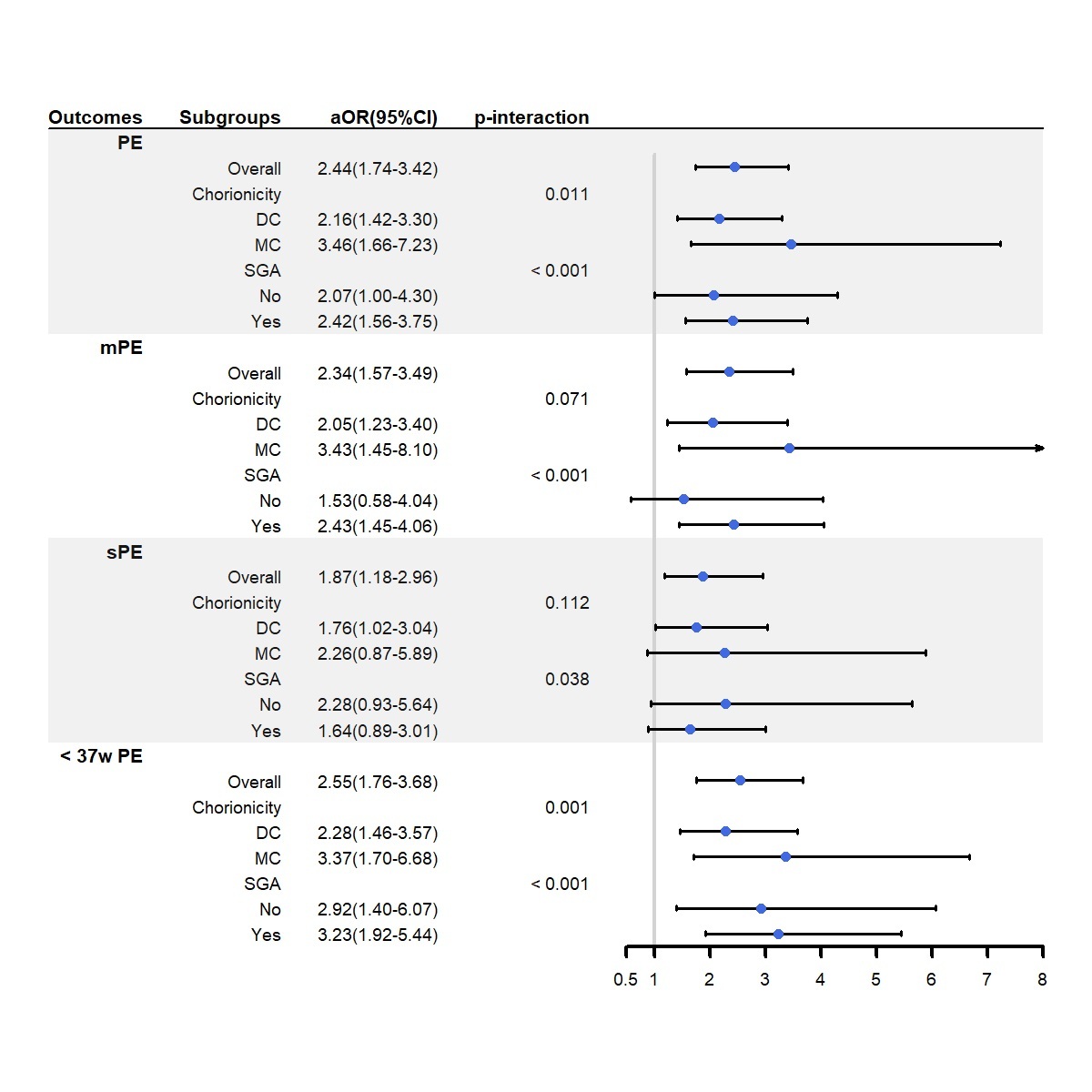

Supplement: Supplementary Figure 2 — The aORs of maternal preeclampsia outcomes of intertwin BWD ≥ 25% in subgroup analysis by SGA and chorionicity. [file Image_2.JPEG]
