# Supplementary material for: Optimal cutoffs of growth discordance for the risk of preeclampsia in twin pregnancies: A single-center retrospective cohort study
Source: Front Cardiovasc Med. 2023 Jan 16;9:1073729. doi: 10.3389/fcvm.2022.1073729 (PMC9884673; doi:10.3389/fcvm.2022.1073729)
Supplement: Supplementary file 3 [file Table_1.DOCX]

**Supporting information**

**Table S1.** Comparison of the women included and excluded

**Table S2.** Distributions of variables with missing data to compare the complete case data observed with the results from the datasets pooled with imputed variables from multiple imputation

**Table S3.** The aORs of maternal GH-PE outcomes of different degrees of intertwin BWD in complete cases

**Table S1. Comparison of the women included and excluded**

| **Variables** | **Statistic** | **N with  observed data  from included** | **Included in study** | **N with  observed data from  potentially eligible** | **Potentially eligible** |
| --- | --- | --- | --- | --- | --- |
| Maternal age | Mean±SD | 2332 | 31.0±4.1 | 2402 | 31.0±4.2 |
| Nulliparous | n(%) | 2535 | 2116(83.5) | 2609 | 2175(83.4) |
| ART | n(%) | 2631 | 1352(51.4) | 2705 | 1382(51.1) |
| History of abnormal pregnancy | n(%) | 2631 | 46(1.7) | 2705 | 47(1.7) |
| GDM | n(%) | 2631 | 540(20.5) | 2705 | 559(20.7) |
| Gestational age at delivery (wk) | Mean±SD | 2631 | 35.7±2.0 | 2705 | 35.6±2.1 |
| MC twin | n(%) | 2050 | 448(21.9) | 2107 | 480(22.8) |
| Cesarean delivery | n(%) | 2625 | 2487(94.7) | 2698 | 2550(94.5) |
| Average birthweight (g) | Mean±SD | 2631 | 2462.8±425.4 | 2705 | 2444.5±443.3 |
| Larger twin birthweight (g) | Mean±SD | 2631 | 2606.6±447.4 | 2705 | 2591.0±460.9 |
| Smaller twin birthweight (g) | Mean±SD | 2631 | 2319.0±435.2 | 2705 | 2297.9±460.1 |
| SGA in at least one twin | n(%) | 2631 | 841(32.0) | 2705 | 869(32.1) |

*SD, standard deviation; ART, assisted reproductive technology; GDM, gestational diabetes mellitus; MC, monochorionic; SGA, small for gestational age*

**Table S2. Distributions of variables with missing data to compare the complete case data observed with the results from the datasets pooled with imputed variables from multiple imputation**

| **Characteristics** | **Statistic** | **Number (%) with missing data** | **Complete case** | **Multiple imputation** |
| --- | --- | --- | --- | --- |
| Maternal age | Mean | 299(11.4) | 31.0 | 31.0 |
| Nulliparous | n(%) | 96(3.6) | 2116(83.5) | 2197(83.5) |
| MC twin | n(%) | 581(22.1) | 448(21.9) | 585(22.2) |
| Cesarean delivery | n(%) | 6(0.2) | 2487(94.7) | 2492(94.7) |

*MC, monochorionic*

**Table S3. The aORs of maternal GH-PE outcomes of different degrees of intertwin BWD in complete cases**

| **Outcomes** | **Crude OR(95%CI)** | **p-value** | **Adjusted OR(95%CI)** | **p-value** |
| --- | --- | --- | --- | --- |
| **GH-PE** |  |  |  |  |
| <15% | reference | - | reference | - |
| 15-20% | 0.98(0.70-1.37) | 0.884 | 0.97(0.69-1.36) | 0.857 |
| 20-25% | 1.39(0.92-2.10) | 0.117 | 1.34(0.88-2.03) | 0.169 |
| ≥25% | 2.41(1.65-3.53) | <0.001 | 2.43(1.65-3.58) | <0.001 |
| **GH** |  |  |  |  |
| <15% | reference | - | reference | - |
| 15-20% | 0.89(0.45-1.77) | 0.747 | 0.89(0.45-1.77) | 0.737 |
| 20-25% | 1.97(1.01-3.84) | 0.048 | 1.89(0.96-3.72) | 0.065 |
| ≥25% | 1.60(0.77-3.29) | 0.207 | 1.64(0.79-3.39) | 0.187 |
| **PE** |  |  |  |  |
| <15% | reference | - | reference | - |
| 15-20% | 1.00(0.70-1.45) | 0.985 | 1.00(0.69-1.45) | 0.995 |
| 20-25% | 1.15(0.72-1.83) | 0.568 | 1.11(0.69-1.77) | 0.677 |
| ≥25% | 2.38(1.59-3.55) | <0.001 | 2.38(1.58-3.57) | <0.001 |
| **mPE** |  |  |  |  |
| <15% | reference | - | reference | - |
| 15-20% | 0.81(0.48-1.38) | 0.438 | 0.81(0.48-1.38) | 0.435 |
| 20-25% | 0.72(0.34-1.51) | 0.388 | 0.71(0.34-1.49) | 0.361 |
| ≥25% | 2.66(1.65-4.29) | <0.001 | 2.65(1.63-4.30) | <0.001 |
| **sPE** |  |  |  |  |
| <15% | reference | - | reference | - |
| 15-20% | 1.21(0.76-1.92) | 0.431 | 1.20(0.75-1.91) | 0.450 |
| 20-25% | 1.58(0.80-2.76) | 0.110 | 1.51(0.86-2.65) | 0.154 |
| ≥25% | 1.59(0.91-2.79) | 0.103 | 1.56(0.89-2.75) | 0.121 |
| **<34w PE** |  |  |  |  |
| <15% | reference | - | reference | - |
| 15-20% | 2.52(0.87-7.33) | 0.089 | 2.47(0.85-7.23) | 0.098 |
| 20-25% | 1.91(0.42-8.72) | 0.403 | 2.07(0.45-9.57) | 0.354 |
| ≥25% | 8.11(3.20-20.55) | <0.001 | 8.00(3.09-20.67) | <0.001 |
| **<37w PE** |  |  |  |  |
| <15% | reference | - | reference | - |
| 15-20% | 0.91(0.59-1.41) | 0.672 | 0.91(0.58-1.41) | 0.664 |
| 20-25% | 1.41(0.85-2.34) | 0.182 | 1.37(0.82-2.28) | 0.225 |
| ≥25% | 2.58(1.67-3.98) | <0.001 | 2.48(1.60-3.86) | <0.001 |

Adjusted such as parity, maternal age, delivery mode, assisted reproductive technology, chorionicity, and gestational diabetes mellitus; BWD <15% group taken as reference

*BWD, birthweight difference; OR, odds ratio; CI, confidence interval; GH, gestational hypertension; PE, preeclampsia;mPE, mild preeclampsia; sPE, severe preeclampsia; < 34w PE, preeclampsia delivered before 34 weeks; < 37w PE, preeclampsia delivered before 37 weeks*
